# Bimodal distribution pattern associated with the PCR cycle threshold (*Ct*) and implications in COVID-19 infections

**DOI:** 10.1038/s41598-022-18735-2

**Published:** 2022-08-25

**Authors:** Doris Yang, Donna E. Hansel, Marcel E. Curlin, John M. Townes, William B. Messer, Guang Fan, Xuan Qin

**Affiliations:** 1 Department of Pathology and Laboratory Medicine, Oregon Health & Science University School of Medicine, 3181 SW Sam Jackson Park Road, L-113, Portland, OR 97239 USA; 2 Department of Medicine, Division of Infectious Diseases, Oregon Health & Science University School of Medicine, Portland, OR 97239 USA; 3 Department Molecular Microbiology and Immunology, Oregon Health & Science University School of Medicine, Portland, OR 97239 USA

**Keywords:** Microbiology, Medical research, Mathematics and computing

## Abstract

SARS-CoV-2 is notable for its extremely high level of viral replication in respiratory epithelial cells, relative to other cell types. This may partially explain the high transmissibility and rapid global dissemination observed during the COVID-19 pandemic. Polymerase chain reaction (PCR) cycle threshold (*Ct*) number has been widely used as a proxy for viral load based on the inverse relationship between *Ct* number and amplifiable genome copies present in a sample. We examined two PCR platforms (Centers for Disease Control and Prevention 2019-nCoV Real-time RT-PCR, Integrated DNA Technologies; and TaqPath COVID-19 multi-plex combination kit, ThermoFisher Scientific) for their performance characteristics and *Ct* distribution patterns based on results generated from 208,947 clinical samples obtained between October 2020 and September 2021. From 14,231 positive tests, *Ct* values ranged from 8 to 39 and displayed a pronounced bimodal distribution. The bimodal distribution persisted when stratified by gender, age, and time period of sample collection during which different viral variants circulated. This finding may be a result of heterogeneity in disease progression or host response to infection irrespective of age, gender, or viral variants. Quantification of respiratory mucosal viral load may provide additional insight into transmission and clinical indicators helpful for infection control.

## Introduction

Since the onset of the COVID-19 pandemic in late 2019, SARS-CoV-2 has been distinguished by its unprecedented transmissibility compared to related coronaviruses such as SARS-CoV and MERS CoV. By March 11, 2020 when the World Health Organization declared COVID-19 a pandemic, there had already been over 118,000 cases in 114 countries and 4291 deaths (WHO)^1^; these tallies have ballooned to cumulative totals of approximately 532 million cases and 6.3 million deaths worldwide as of June 7, 2022. The amount of virus produced at the respiratory epithelium is considered to be a critical element in disease^2,3^, though not the only factor in determining SARS-CoV-2 transmissibility^4,5^. Viral RNA load has also been investigated as a possible correlate of severity of illness^6,7^, host cell type or specific anatomic site of intense viral replication^5^, viral replication dynamics during the course of clinical illness^8^, and inoculum effect or infective dose. Polymerase chain reaction (PCR) cycle threshold (*Ct*) values have also been widely referenced as both epidemiological indicators and clinical indicators of disease burden and outcomes^9,10^.

A common technique for measuring SARS-CoV-2 viral load is through quantitative analysis of viral RNA genomic copy numbers. Quantitative viral RNA studies have generally treated the inverse polymerase chain reaction (PCR) cycle threshold (*Ct*) as a proxy for relative levels of viral genomes or viral load^7^. However, many different PCR platforms have been developed for detection of SARS-CoV-2 and most were not originally intended to be fully quantitative. The degree of analytical variability associated with these assays can be minimized in the context of high throughput testing under a robust quality management system, and for this reason clinical laboratories involved in the pandemic response commonly validate multiple PCR platforms with correlation studies on a regular basis^11^.

In this study, we compared the performance characteristics of two leading PCR-based methods for quantification of SARS-CoV-2 targeting multiple viral genomic regions, and sought to characterize the *Ct* value distribution of SARS-CoV-2 RNA over a large sample size and date range. Besides quantifying viral genome copies, we examined *Ct* distribution patterns in order to understand viral replication potentials in host populations, and to develop reporting strategies to improve effectiveness of infection prevention.

## Results

### Test performance and assay dynamic range

**Figure 1:**
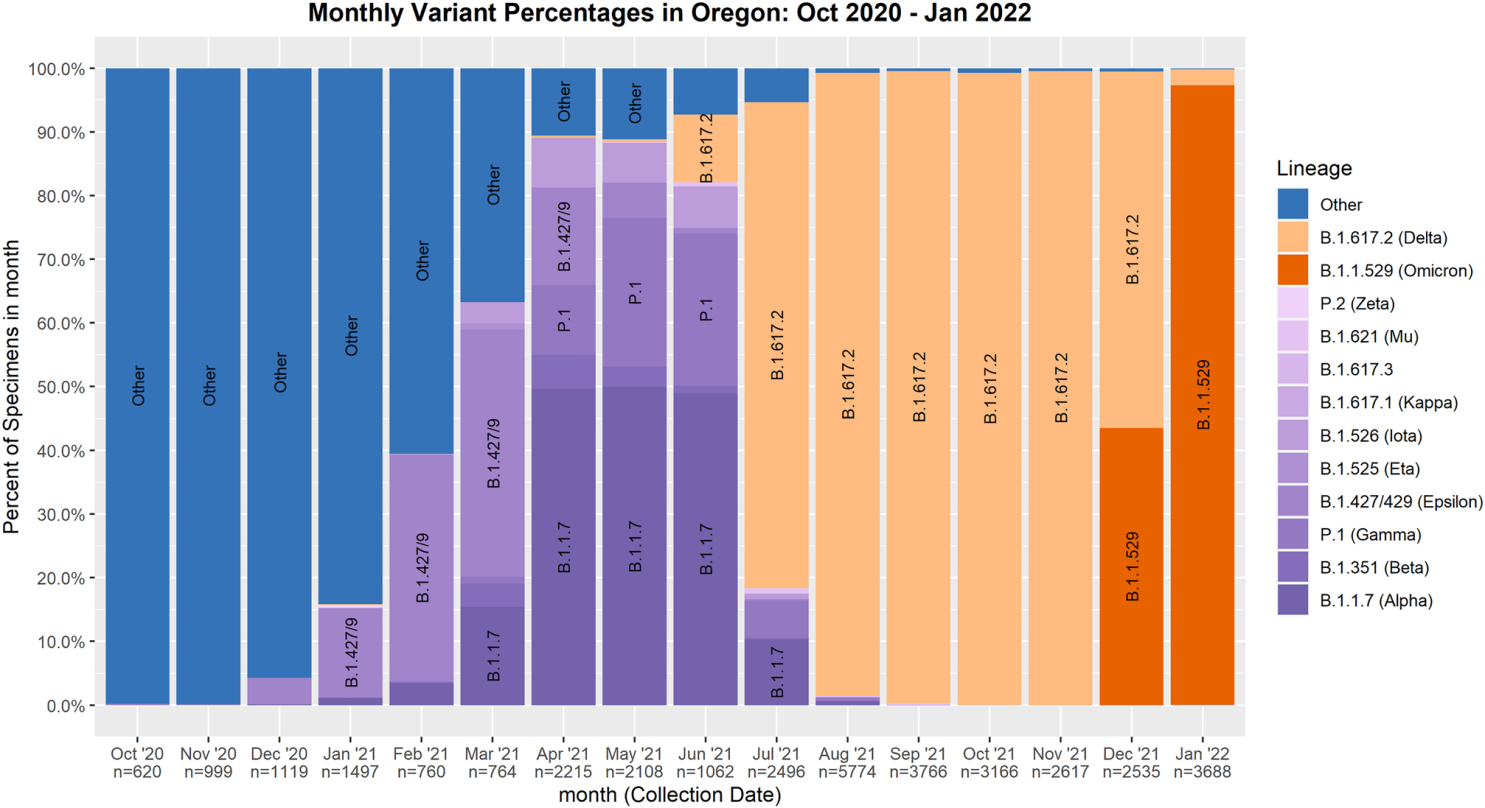
SARS-CoV-2 variant percentages in Oregon, September 2020 to January 2022.

Nucleic acid amplification was performed using either the 2019-nCoV CDC EUA Kit (IDT Integrated DNA Technologies, Inc., i.e., “CDC platform”) or the TaqPath™ Multiplex RT-PCR COVID-19 kit (Thermo Fisher Scientific, Inc., i.e., “Fisher platform”) (see “Methods”). We performed 208,947 PCR tests for SARS-COV-2 during 52 weeks between October 2020 and September 2021 using one of two platforms available (Table 1). In total, 14,231 (6.8%) positive tests were resulted and associated with 13,553 individuals. Patients contributing to the positive dataset ranged in age from 2 h to 103 years; 48% were female. Most (97%) of the positive tests resulted from persons presenting for clinical care. Positive tests also included 185 of 37,908 (0.5% positive rate or 1.3% of the total positive findings) pre-operation (pre-op) screening tests, and 589 of 23,262 (2.5% positive rate or 4.1% of the total positive findings) healthcare staff tests. The prevailing variants of concern evolved over the time period of the study, as reflected by GISAID data summary for Oregon (Fig. 1). In addition, we included 878 test results between December 6, 2021 through January 22, 2022 that were positive but failed to amplify the S gene (S gene target failure or SGTF) with the Fisher PCR platform, in order to include likely omicron variants.

**Table 1:** Bimodality Coefficients and Hartigan’s Dip Test p-values of PCR Ct values by age and gender of patients and date of collection (values shaded in grey do not meet the threshold for Bimodality Coefficient or multimodality by Hartigan’s Dip Test).

	Hartigan’s Dip Test p-value		Bimodality coefficient	
	N1	N2	N1	N2
**CDC PCR chemistry**				
All CDC (n = 5212) Oct 2020–Sep 2021	< 0.001	< 0.001	0.5574	0.5725
Age (Oct 2020–Sep 2021)				
65+ (n = 350)	0.0069	0.0030	0.5916	0.6079
21–64 (n = 3739)	< 0.001	< 0.001	0.5631	0.5756
< 21 (n = 1028)	< 0.001	< 0.001	**0.5403**	0.5602
< 17 (n = 655)	< 0.001	< 0.001	**0.5213**	**0.5402**
< 12 (n = 376)	0.0386	0.0293	**0.5238**	**0.5392**
< 5 (n = 100)	**0.0561**	0.0107	0.5560	0.5783
Gender (Oct 2020–Sep 2021)				
Female (n = 2338)	< 0.001	< 0.001	0.5557	0.5716
Male (n = 2520)	< 0.001	< 0.001	0.5695	0.5842
Date range				
Oct–Dec 2020 (n = 3149)	< 0.001	< 0.001	**0.5497**	0.5687
Jan–Sep 2021 (n = 2063)	< 0.001	< 0.001	0.5719	0.5782

	N	ORF1ab	N	ORF1ab
**Fisher PCR chemistry**				
All Fisher (n = 8460) Oct 2020–Sep 2021	< 0.001	< 0.001	**0.5067**	**0.5267**
Omicron (n = 878)*	0.0100	0.0201	**0.4851**	**0.5156**

Numeric values that were below the statistic cut-off values are given in bold.

*S gene target failure by Fisher multiplex PCR chemistry.

The viral load range observed in this analysis was very broad with *Ct* values ranging from < 8 to 39. To reduce signal noise for pattern analysis, we removed all extreme values beyond the expected linear range (*Ct* ≤ 9, or ≥ 39). We removed 353 Fisher samples and 214 CDC samples based on these criteria. The resulting *Ct* span of 10–38 corresponds to absolute viral genomic copies ranging from approximately 1.5 × 10^10^–1.5 × 10^2^, where every 3 cycles constitutes roughly a one log viral titer change^12,13^.

**Figure 2:**
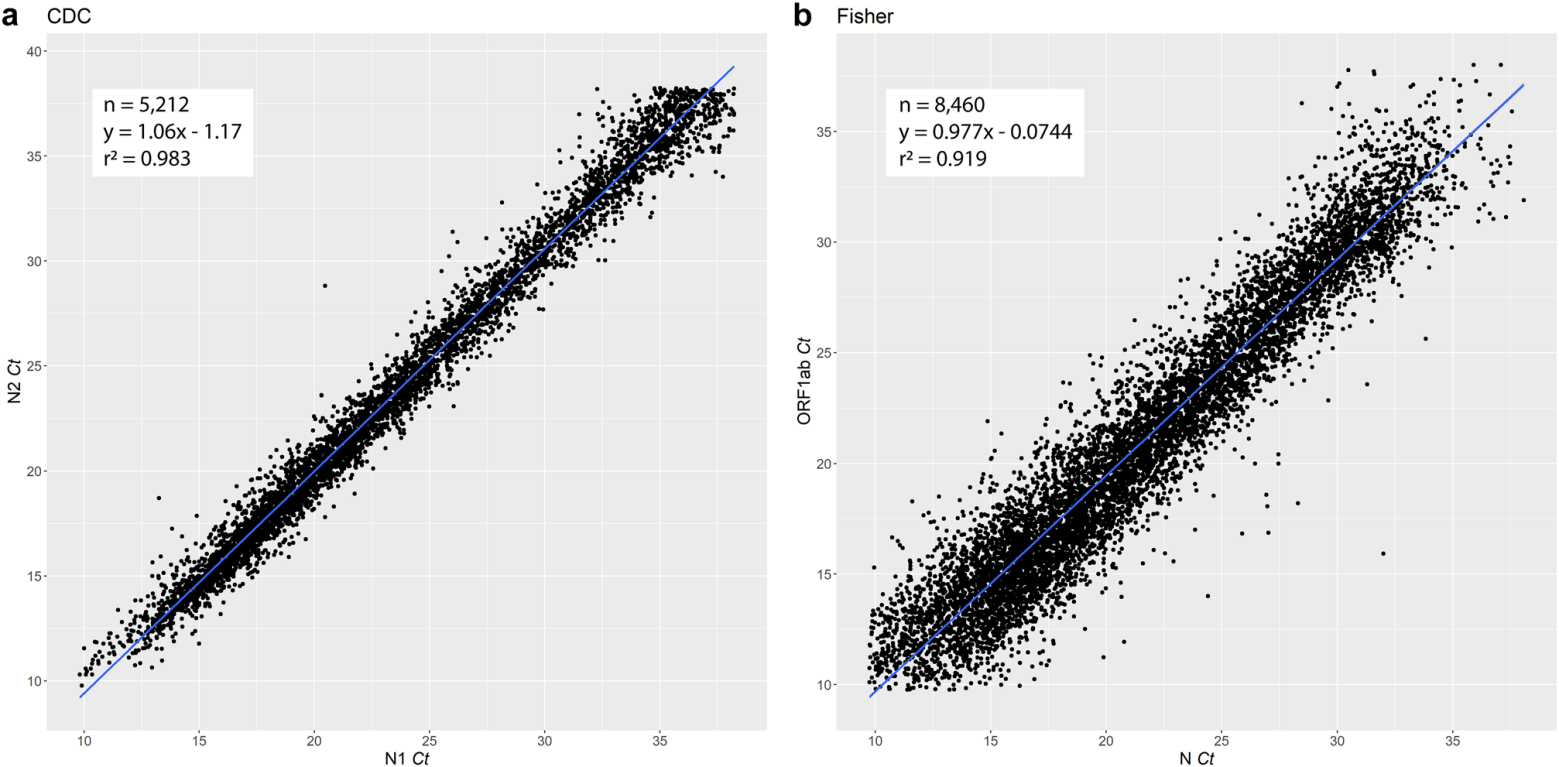
Correlation of PCR *Ct* distribution between two viral targets associated with CDC or Fisher chemistry.

We performed an intra-assay *Ct* comparison between the N1 and N2 targets in the CDC platform, and the N and ORF1ab targets in the Fisher platform. Highly linear relationships between *Ct* values from two independent viral targets employed was observed in both platforms (Fig. 2) with coefficients of determination (R^2^) of 0.983 and 0.919 respectively. Given the slightly higher correlation performance of the CDC PCR platform, we proceeded to focus on our subgroup analysis of *Ct* distribution on samples tested with this method.

### Analysis of bimodal distribution of *Ct* values

**Figure 3:**
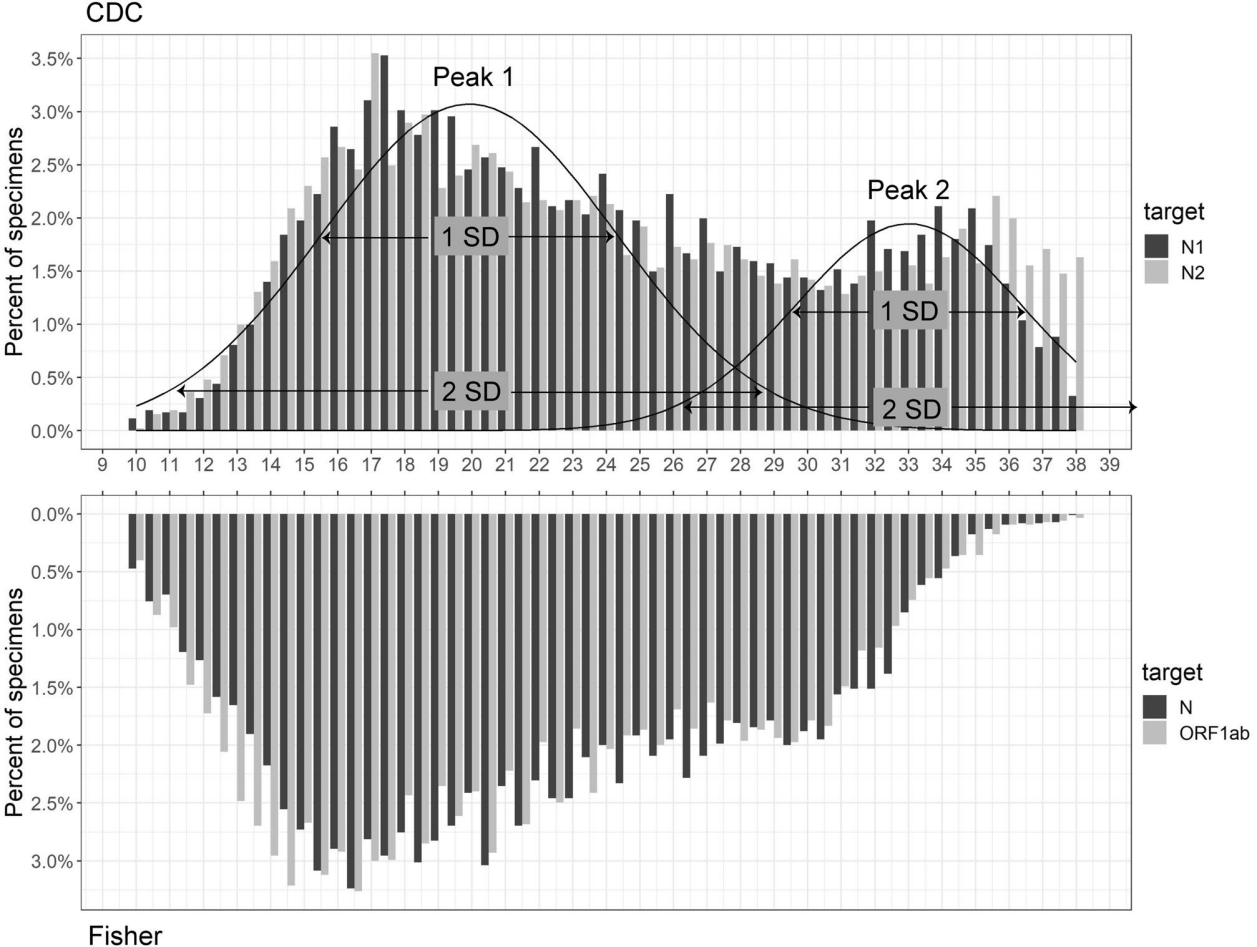
PCR *Ct* distribution of N1 and N2 targets by CDC chemistry and N and ORF1ab targets by Fisher chemistry.

Initial plotting of the *Ct* values for both the N1 and N2 targets of the 5214 CDC PCR samples revealed a non-normal distribution (Fig. 3), thus rendering traditional summary statistics ill-suited and insufficient for describing the distribution. In this analysis, Hartigan’s Dip Test (https://CRAN.R-project.org/package=diptest) was used to confirm non-normal multimodality while the Bimodality Coefficient Test was used to specifically capture the observed bimodality. The p-value of Hartigan’s Dip Test was < 2.2e − 16 for both platforms regardless N1 and N2, or N and ORF1ab, in the 52-week period evaluation. This test alone confirmed the alternative hypothesis of a non-unimodal *Ct* number distribution for both platforms. The Bimodality Coefficient was 0.557 for the N1 target and 0.565 for the N2 target; both were above the critical value of 0.555, indicating a bimodal distribution for the CDC platform (Table 1). While *Ct* numbers generated from samples tested by Fisher platform also appeared to form two peaks, both N and ORF1ab targets failed Bimodality Coefficient tests (Table 1 and Fig. 3). The main difference between the two platforms was the PCR design by CDC single-plex versus Fisher multiplex. Thus, the examination of bimodality against viral or host factors in subgroup analysis was carried out using samples tested by CDC platform only.

**Figure 4:**
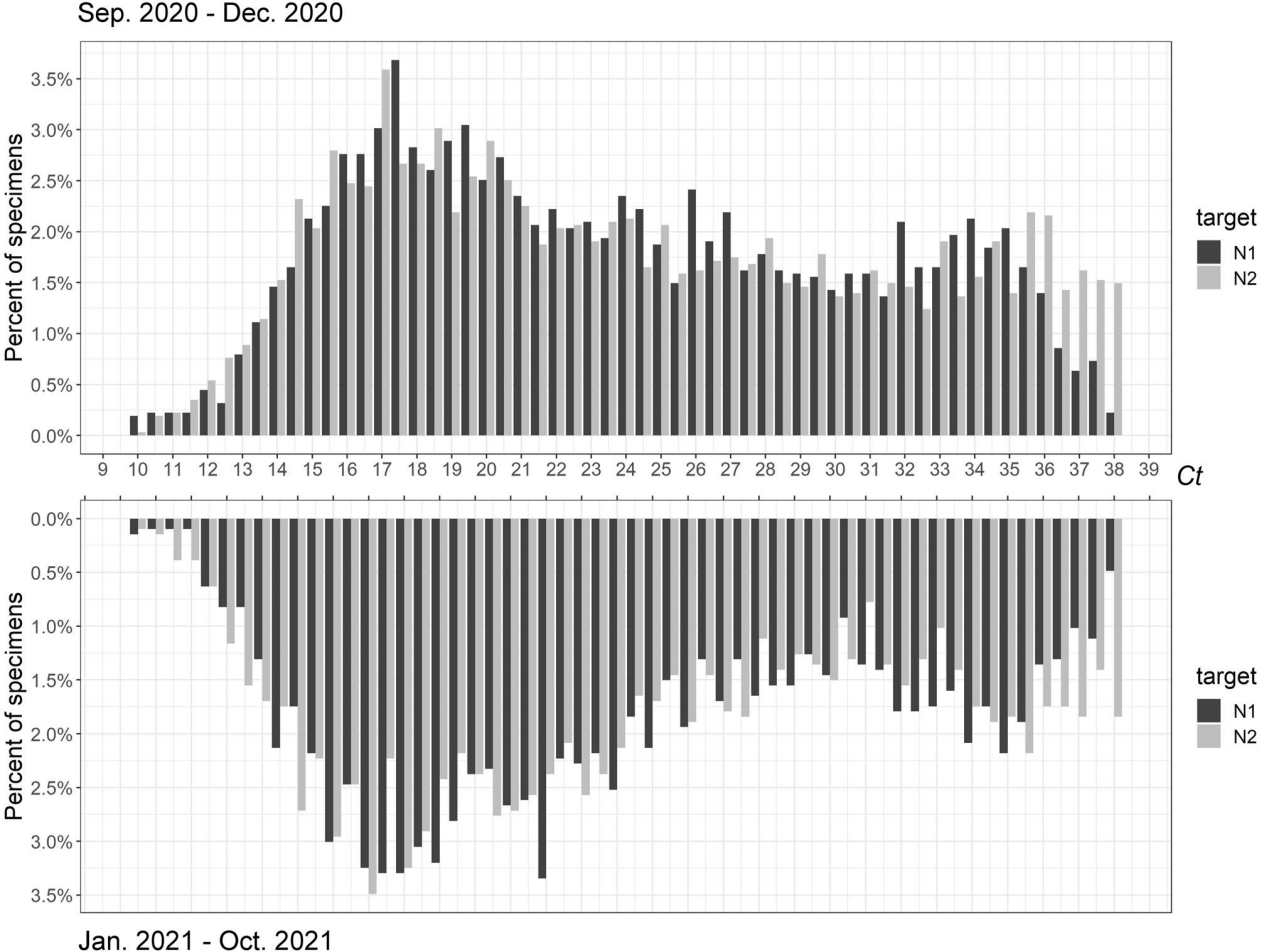
PCR *Ct* distribution of N1 and N2 targets from samples collected in 2020 and 2021.

The bimodal *Ct* distribution pattern was further examined by viral variant and demographic factors. To evaluate the impact of variants and vaccinations, we compared the *Ct* distributions separated by time frame: 2020 versus 2021 coinciding with the repeated emergence of SARS-CoV-2 variants of concern in 2021 (Table 1, Figs. 1 and 4). The bimodal pattern associated with either 2020- or 2021-time frame remained stable, passing both Hartigan’s Dip Test and Bimodality Coefficient requirements with the exception of N1 falling below the benchmark of 0.555 in 2020 (Table 1). In a sub-analysis, we included 878 Fisher PCR results from December 6, 2021 to January 15, 2022 that showed the suspected omicron pattern of SGTF. Although only the first 109 samples of the 878 were confirmed omicron by full genomic sequencing at the time of manuscript writing, we again observed two peaks in *Ct* distribution for both N and ORF1ab which were confirmed by Hartigan’s Dip Test, but not by the Bimodality Coefficient test (Table 1 and Suppl Fig. 1).

**Figure 5:**
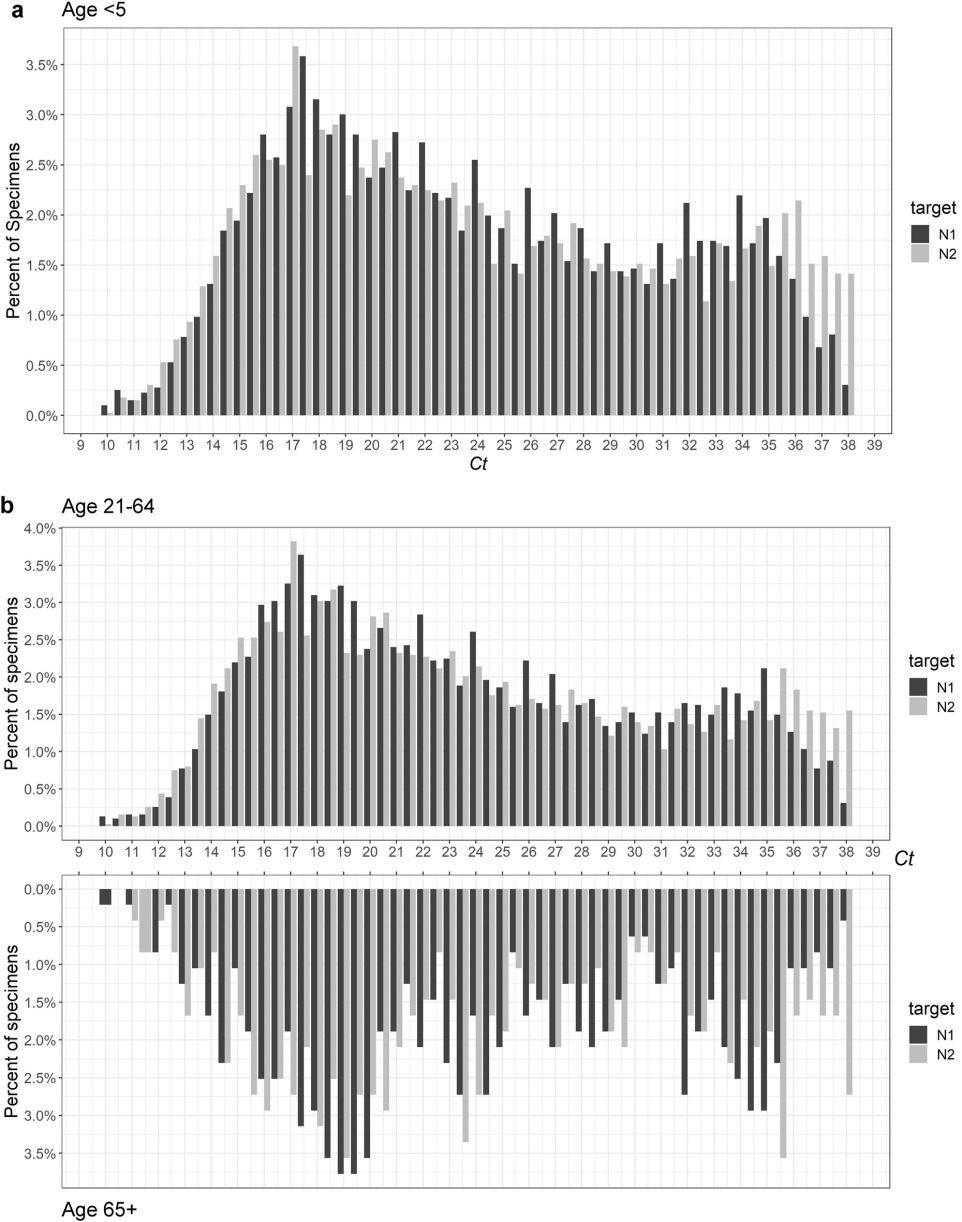
PCR *Ct* distribution of N1 and N2 associated with age groups: (**a**) age < 5 years, (**b**) age groups of 21–64 years and > 65 years.

**Figure 6:**
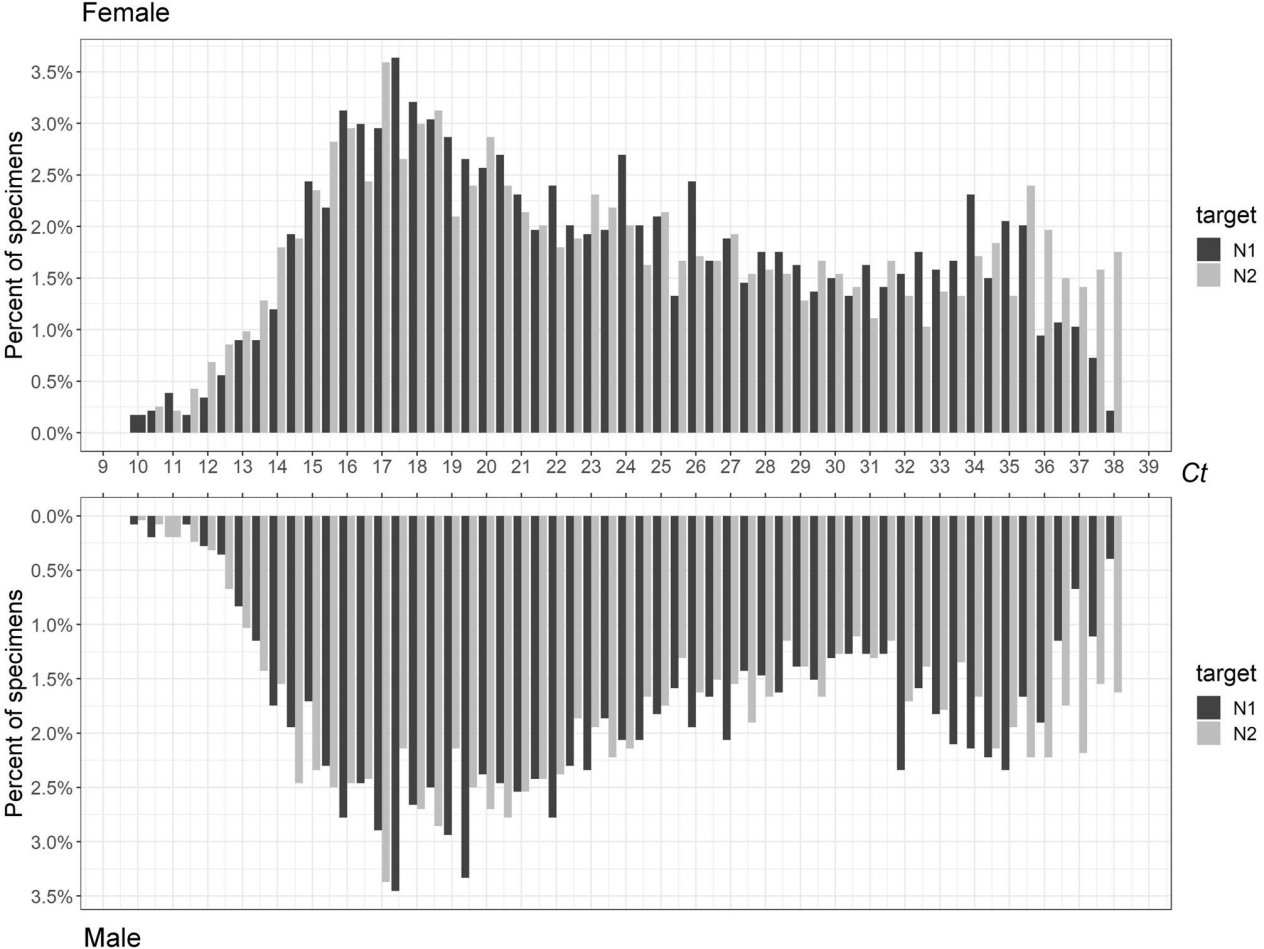
PCR *Ct* distribution of N1 and N2 targets from samples associated with female and male patients.

Finally, we evaluated the impact of age and gender (Table 1 and Figs. 5, 6, Suppl Fig. 2). Both female and male subgroups displayed bimodality, passing both Hartigan’s Dip Test and Bimodality Coefficient requirements (Table 1 and Fig. 6). The adult (21–64 years) and older adult (65+ years) cohorts displayed bimodality according to both tests (Table 1 and Fig. 5). We examined multiple subgroups within the child and adolescent cohort to consider varying exposure ranges resulting from developmentally marked differences in behavioral, social, and travel patterns associated with subgroups. In particular, we analyzed < 5 years, < 12 years, < 17 years, and < 21 years subgroups corresponding roughly to pre-school, elementary, middle-high school, and college students (Table 1 and Fig. 5, Suppl Fig. 2). Overall, children and adolescent age cohorts had less definitive bimodality as compared to the 21–64 and 65+ age cohorts. While the < 17 and < 12 age cohorts failed the Bimodality Coefficient test for both targets, both are not unimodal per Hartigan’s Dip Test (Table 1 and Suppl Fig. 2). Meanwhile, the < 5 age cohort displayed stronger bimodality, with the only failed test being a N1 target p-value slightly above the cutoff for Hartigan’s Dip Test (Table 1 and Fig. 5). In general, more sample groups generated by the CDC platform passed bimodality examinations by Hartigan’s Dip Test as most of all the p-values remained below 0.05 (Table 1). Notably, the Bimodality Coefficient test appeared to be more stringent than Hartigan’s Dip test, indicative of its specificity for bimodality confirmation.

### Bimodal deconvolution and characterization of contributing populations

Bimodal distributions frequently arise when a dataset is composed of two contributing populations corresponding to alternative values of a binary parameter. Under this assumption, some information about each contributing population can be inferred. After establishing bimodality in our distribution of *Ct* values, we quantitively measured the two peaks by fitting a mixture of two normal distributions^14^. This yielded a mean of ~ 20 for the first *Ct* peak and a mean of ~ 33 for the second *Ct* peak (Table 2). The mixing proportions, or lambdas, show that approximately 30% of samples fall under the first peak. Taking the upper bounds of 0.5 or 1.0 standard deviation range of the first peak, approximately 47% of the samples fall under the *Ct* of 22 and 56% fall under the *Ct* of 24, respectively (Table 2). It was possible that our fitting method may have introduced a bias to the peaks as the first peak appeared to be right shifted and the second peak to be left shifted (Fig. 3).

**Table 2:** Statistics for fitting of two-component normal mixture distribution for PCR *Ct* values by CDC Chemistry.

PCR target		Peak 1 (% of samples, *Ct* < upper bound of SD)	Peak 2
**N1**	Mixing proportion	0.6791	0.3209
	Mean	20.0	32.5
	SD	4.18	3.16
	0.5 SD range	18.0–22.0 (47%, Ct < 22)	30.5–34.0
	1 SD range	16.0–24.0 (56%, Ct < 24)	29.0–35.5
	2 SD range	11.5–28.5 (70%, Ct < 28)	26.0–38.5
**N2**	Mixing proportion	0.6718	0.3282
	Mean	20.0	33.0
	SD	4.36	3.37
	0.5 SD range	17.5–22.0 (47%, Ct < 22)	31.5–34.5
	1 SD range	15.5–24.5 (55%, Ct < 24)	29.5–36.5
	2 SD range	11.0–28.5 (70%, Ct < 29)	26.5–39.5

## Discussion

In this analysis of 14,231 clinical SARS-CoV-2 PCR tests, two mainstream platforms for amplification and quantification of viral gene targets displayed similar analytic characteristics. Both the CDC and the Fisher platforms produced highly linear *Ct* correlations with coefficient of determination close to 1 between their corresponding two viral targets (N1 and N2, or N and ORF1ab) used. Moreover, these data confirm previous findings showing an extremely wide range of PCR *Ct* values in nasopharyngeal swab samples (*Ct* range 8–39, Fig. 1), with corresponding viral titers ranging from a few copies to billions of copies. When *Ct* distribution patterns were examined, samples appeared to depart from normality to form two peaks along the *Ct* gradients in each of the platforms used (Fig. 3). However, the separation of the two peaks was less pronounced with the Fisher platform, possibly due to the multiplex format and consequently competitive nature of target amplification in this assay, which would be expected to result in reduced amplification efficiency and right-shifting of the distribution peak^14^. Upon further analysis of data from the CDC platform, the observed *Ct* distribution pattern was independent of patient age, gender, and time period of sample collection, during which a number of different variants were predominant.

Notably, the distribution of *Ct* values observed in our series was bimodal (Table 1 and Figs. 3, 4, 5), suggesting contribution from two distinct subsets of samples. This effect is likely not an artifact of sample quality or preparation. The potential contributory factor pertaining to sample quality variation to the bimodality *Ct* distribution is ruled out as CDC platform included host *RNase* P as an internal control^15^. Previous studies have demonstrated that viral titer can be associated with inoculum size, tropism or replication in specific tissue or cell types, and risk of onward transmission^5^. Viral titers derive clinical significance from their possible association with disease severity and/or outcome^16–19^ and their likely correlation with transmissibility^20,21^. Importantly, high levels of viral shedding may occur in asymptomatic hosts, posing substantial challenges to infection control efforts^22,23^. However, there is currently little published information on COVID-19 *Ct* value distribution patterns or their significance to virus-host interactions in SARS-CoV-2 infection. A few studies that did note *Ct* distribution properties outside of normality did not analyze its significance in microbial and host relations distinctively associated with SARS-CoV-2^8,24^. We explored whether the pattern of viral levels at the population level could provide insight into the nature of SARS-CoV-2 replication and shedding difference potentially useful for infection prevention.

When the *Ct* distribution pattern was examined by age groups, the heterogeneous non-unimodal distribution was evident. For age groups of < 5, < 21 (by N2 only), 21–64, and 65+ years, their *Ct* distributions have met the bimodality coefficient criteria (Table 1). However, the non-bimodal nor unimodal *Ct* distribution pattern associated with the age groups of < 12 and < 17 years remains puzzling, when that of age group < 5 years was clearly bimodal. This result suggests there may be underlying differences between viral replication in very young patients vs teens. Otherwise, the bimodal nature of the *Ct* distribution was unaffected by gender, or calendar time-period, during which several different variants predominated. Notably, the *Ct* distribution of the 878 omicron samples appeared to show the two positive peaks skewing closer into each other (Supplemental Fig. 1). The putative Omicron *Ct* distribution curves failed Bimodality Coefficient test. It is again possible that the Fisher multiplex chemistry suppressed the expression of bimodality as seen in the overall 52-week analysis (Table 1). However, we believe there is still sufficient evidence to support the finding of this dichotomous distribution of viral replication pattern in the host population. More studies using other test platforms are needed to confirm this finding.

Host factors must play a role in heterogeneous viral replication properties. SARS-CoV-2 cell entry is mediated by human angiotensin-converting enzyme II (*ACE2*) and *ACE2* polymorphisms, which may affect the risk for SARS-CoV-2 infection and the course of COVID-19^25^. In a multivariable analysis by Nikiforuk et al., the researchers showed that the greatest viral RNA loads were observed in participants with high transmembrane *ACE2* transcription, while transcription of the soluble isoform appears to protect against high viral RNA load in the upper respiratory tract^26^. It is possible that multiple host genetic factors, innate and adaptive immunity, and respiratory microbiota may all play roles in viral titers and disease outcomes^27,28^.

The wide range of *Ct* values and corresponding viral loads in our study supports the notion that SARS-CoV-2 transmission occurs heterogeneously^23,29^. It stands to reason that high viral load carriers likely contribute most to new transmissions in the community. An operational categorization separating high from low/moderate viral shedding could therefore be relevant to isolation requirements after infection, and infection control efforts. Using a cutoff value of *Ct* < 22–24, corresponding to the upper bound of 0.5 SD–1 SD of the first peak, representing 47–56% of individuals in this cohort, could be used as indicators separating levels of respiratory tract viral shedding potentials. Ideally any categorization would be tested against presence of culturable virus and risk of transmission in clinical studies.

There are several limitations of this analysis. Notably, the asymptomatic population is less likely to be well represented in our study population as both pre-operative and staff screenings contributed far fewer positives (5.4% combined) than other groups. This sampling bias prevented us from any speculations over the differences between symptomatic versus asymptomatic which may contribute to bimodality. Although all samples were collected by healthcare workers, it is possible that there was variability in sample collection procedure compliance, particularly between age subgroups. This study did not include information on patient clinical course, vaccination status, or immune responses at the time samples were collected. We are therefore unable to explicitly relate *Ct* values with these clinical factors. We can only speculate that our tested population likely sought testing because of symptom presentation or suspected infection exposure. In addition, we do not know the identities of viral strain or variant associated with most of the *Ct* values obtained, and this information would be helpful in formally evaluating the role of infecting variant on *Ct* values. The lack of information regarding the stage of infection at the time of sample collection is the biggest limitation of this study. With the data set size, it is likely that that the samples collectively represent a random distribution along the clinical course of the viral infection. Viral loads can vary depending on disease progression, so it is possible that the two peaks represent subpopulations at different stages of infection. Because we were unable to track patients’ *Ct* values over time, we could not determine whether their viral levels were increasing or decreasing at the time of sample collection. Future studies and clinical applications should monitor changes in patients’ *Ct* values through repeat testing.

It is well recognized that PCR *Ct* values and associated viral titers do not correlate well with the intensity of symptoms during SARS-CoV-2 infection, and this information is currently not routinely used in clinical management (https://www.aphl.org/programs/preparedness/Crisis-Management/Documents/APHL-COVID19-Ct-Values.pdf and https://www.idsociety.org/globalassets/idsa/public-health/covid-19/idsa-amp-statement.pdf). However, it is likely that viral titer influences risk of transmission^30^, and it has been suggested that those presenting with higher *Ct* values may require shorter periods of isolation to prevent onward transmission^31^. Our study suggests that patients can be categorized into high and low titer subpopulations at the time of testing. Given the important contribution of super-spreading events to SARS-CoV-2 transmission, this dichotomous *Ct* distribution could therefore provide a relatively simple indicator that might be useful for infection control purposes. For example, a notation indicating *Ct* less than 22–24 (corresponding to viral titers in the millions) be considered^12,13^. Risk-based criteria for isolation and quarantine incorporating viral titer assessments would need to be developed before a reporting notation can be implemented. As the SARS-CoV-2 epidemic continues and new variants emerge, testing and reporting strategies should be maximally leveraged to reduce ongoing community transmission in order to control the case growth rate, healthcare burden, and workforce preservation.

## Materials and methods

### PCR cycle threshold (***Ct***) data on SARS-CoV-2 positive specimens.

We performed 208,947 tests between October 4, 2020 and September 30, 2021. An additional 878 suspected Omicron samples was later included by taking the advantage of Fisher PCR platform using S gene target failure (SGTF) as a surrogate marker after September 30, in late 2021^32^. The tested population consisted of patients who sought testing at OHSU healthcare and community testing facilities as well as patients enrolled in pre-operative screening tests. All samples were collected by qualified healthcare professionals. The specimen type included primarily nasopharyngeal (NP) swabs (> 99.9%), and a small number of laboratory-validated bronchoalveolar lavage samples, tracheal aspirates, nasal swabs and throat swab specimens. All samples with positive PCR results were included in the positive dataset (n = 14,231), and included repeat testing in some individuals. Fewer than 1% of positive samples came from pre-operative screening tests. Samples with “negative” or “inconclusive” PCR results were excluded from the dataset. Excel Microsoft 360 and Tableau 2021.1 were used for data analysis and visualization.

This study was approved by the Oregon Health & Science University Institutional Review Board (STUDY00021396: Collection and archiving of residual nasopharyngeal swab, sputum, and blood samples from persons tested for SARS-CoV-2 infection). This study filed for “Application & Certification for Waiver or Alteration of the HIPAA Authorization Requirement” and the informed consent waiver was approved by the Research Integrity Office of Oregon Health & Science University on May 26, 2020. The OHSU Institutional Review Board (FWA00000161; IRB00000471) complies with United States Federal research guidelines 45 CFR Part 46, 21 CFR Parts 50 and 56, and other federal and Oregon laws and regulations, as applicable. The OHSU IRB also complies with ICH-GCP (E6) codes 3.1–3.4, which outline responsibilities, composition, functions, and operations, procedures, and records of the IRB.

### Viral titers inferred by PCR cycle threshold analysis

RNA extraction was performed by two different methods including MagNA Pure 96 and KingFisher Flex (ThermoFisher Scientific) for this study period, according to manufacturer instructions. To expand test capacity after September, 2020, RNA samples were tested by two PCR platforms: the 2019-nCoV CDC EUA Kit containing N1, N2 and human *RNase* P (RP) primer/probe mix (IDT Integrated DNA Technologies, Inc.) and the TaqPath™ Multiplex RT-PCR COVID-19 kit containing N gene, S gene, ORF1ab primers, and MS2 Phage control (ThermoFisher Scientific). Inter-analyzer correlation studies with regard to RNA extraction and PCR platforms were carried out at the time of initial test validation of RNA, then once every 6 months regularly. Each sample was tested by one or the other platform, but not both. All PCR amplifications were performed using QuantStudio5 thermocyclers (ThermoFisher Scientific) at the limit of detection ~ 15 copies per PCR reaction for all mixed use of RNA extraction and PCR platforms^33^. For data clarity, all PCR *Ct* records in this study were rounded to the nearest 0.5 from their original records containing two decimal points. The corresponding *Ct* distributions of each test were measured by linearity and R-squared goodness-fit test.

A standard curve relating *Ct* values to viral copies ranging from 15 to 500,000 copies per PCR reaction was developed using serial dilutions of a synthetic SARS-CoV-2 RNA control provided by the CDC (data not shown)^7,34^. This confirms that a 3-point change in *Ct* value is roughly equal to a tenfold change in the quantity of the template viral material^12,13^. Similar to other studies^5,7,34^, at viral titers near the limit-of-detection (LOD) using 10 PCR reaction replicates, the *Ct* values are often spread around 33–38, indicative of analytical stochasticity and loss of linearity (data not shown).

### Statistical analysis

We compared performance characteristics between the two PCR test methods using corresponding obtained *Ct* values. We compared 5212 *Ct* values produced by CDC PCR platform to 8460 *Ct* values produced by Fisher PCR platform. The data collection period included several months when the viral variant B.1.1.7 was highly prevalent. B.1.1.7 samples were observed to be associated with S gene target failure when tested using the Fisher PCR platform, and therefore, *Ct* values generated on S gene amplification by multiplex PCR chemistry were not included in analysis. We did not have access to age data for 97 individuals and gender data for 356 individuals, and therefore these samples were excluded from any relevant analyses.

The reliability of the CDC versus Fisher platforms was compared through a linear regression analysis using R.4.1.0 (R Core Team, 2021, https://www.R-project.org/). The bimodality of the CT value distribution was evaluated in two ways: (1) Bimodality Coefficient^35^ and (2) p-value for Hartigan’s Dip Test (https://CRAN.R-project.org/package=diptest). To calculate the Bimodality Coefficient, skewness (m_3_) and kurtosis (m_4_) were first found using the *e1071* R package^36^ (v1.7–7; https://CRAN.R-project.org/package=e1071). These values, along with sample size (n) were substituted into the formula^35^

$$BC= \frac{{m}_{3}^{2}+1}{{m}_{4}+3 \cdot \frac{{(n-1)}^{2}}{(n-2)(n-3)}}$$

The p-value for Hartigan’s Dip Test was calculated using the *diptest* (v0.76-0; Maechler, 2021) R package. When p-value indicates a statistical significance (e.g. p < 0.05) by Hartigan’s test, the distribution is anything but unimodal. Normal mixture modelling was done using the *mclust* (v5.4.9, 2021 R package)^37^. R, Microsoft Excel, and Tableau were also used as tools for visualization.

### Ethics oversight

This study was conducted in accordance with the approved guidelines, and protocols were approved by the Oregon Health & Science University Institutional Review Board (STUDY00021396: Collection and archiving of residual nasopharyngeal swab, sputum, and blood samples from persons tested for SARS-CoV-2 infection). This study filed for “Application & Certification for Waiver or Alteration of the HIPAA Authorization Requirement” and the informed consent waiver was approved by the Research Integrity Office of Oregon Health & Science University on May 26, 2020.

## Supplementary Information

Supplementary Figures.

## Data Availability

The supplemental materials included only two supplemental figures: Supplemental Figs. 1 and 2.

## References

[1] Lewis D (2021). Superspreading drives the COVID pandemic—And could help to tame it. Nature.

[2] Marks M (2021). Transmission of COVID-19 in 282 clusters in Catalonia, Spain: A cohort study. Lancet Infect Dis..

[3] Spinelli MA, Rutherford G, Gandhi M (2021). Lowering SARS-CoV-2 viral load might affect transmission but not disease severity in secondary cases—Authors' reply. Lancet. Infect. Dis..

[4] Puhach O (2022). Infectious viral load in unvaccinated and vaccinated patients infected with SARS-CoV-2 WT, Delta and Omicron. medRxiv..

[5] Liu J (2021). SARS-CoV-2 cell tropism and multiorgan infection. Cell Discov..

[6] Salto-Alejandre S (2021). SARS-CoV-2 viral load in nasopharyngeal swabs is not an independent predictor of unfavorable outcome. Sci. Rep..

[7] Bland J, Kavanaugh A, Hong LK, Kadkol SS (2021). Development and validation of viral load assays to quantitate SARS-CoV-2. J. Virol. Methods.

[8] Jones TC (2021). Estimating infectiousness throughout SARS-CoV-2 infection course. Science.

[9] van Kampen JJA (2021). Duration and key determinants of infectious virus shedding in hospitalized patients with coronavirus disease-2019 (COVID-19). Nat. Commun..

[10] Yin N (2021). Leveraging of SARS-CoV-2 pcr cycle thresholds values to forecast COVID-19 trends. Front. Med..

[11] Binnicker MJ (2020). Challenges and controversies to testing for COVID-19. J. Clin. Microbiol..

[12] Rabaan AA (2021). Viral dynamics and real-time RT-PCR Ct values correlation with disease severity in COVID-19. Diagnostics (Basel)..

[13] Tom MR, Mina MJ (2020). To interpret the SARS-CoV-2 test, consider the cycle threshold value. Clin. Infect. Dis..

[14] Chifiriuc, M. C. *et al.* in *Food Preservation* (ed. A.M. Grumezescu) 645–669 (Academic Press, 2017).

[15] Kanji JN (2021). False negative rate of COVID-19 PCR testing: A discordant testing analysis. Virol. J..

[16] Jacot D, Greub G, Jaton K, Opota O (2020). Viral load of SARS-CoV-2 across patients and compared to other respiratory viruses. Microbes Infect..

[17] Glenet M (2021). Asymptomatic COVID-19 adult outpatients identified as significant viable SARS-CoV-2 shedders. Sci. Rep..

[18] Faico-Filho KS, Passarelli VC, Bellei N (2020). Is higher viral load in SARS-CoV-2 associated with death?. Am. J. Trop. Med. Hyg..

[19] Wright J (2021). Cycle threshold values are inversely associated with poorer outcomes in hospitalized patients with COVID-19: A prospective, observational cohort study conducted at a UK tertiary hospital. Int. J. Infect. Dis..

[20] Kawasuji H (2020). Transmissibility of COVID-19 depends on the viral load around onset in adult and symptomatic patients. PLoS ONE.

[21] Lyngse FP (2021). Increased transmissibility of SARS-CoV-2 lineage B.1.1.7 by age and viral load. Nat. Commun..

[22] Beldomenico PM (2020). Do superspreaders generate new superspreaders? A hypothesis to explain the propagation pattern of COVID-19. Int. J. Infect. Dis..

[23] Yang Q (2021). Just 2% of SARS-CoV-2-positive individuals carry 90% of the virus circulating in communities. Proc. Natl. Acad. Sci. USA..

[24] Young RM (2021). Smartphone screen testing, a novel pre-diagnostic method to identify SARS-CoV-2 infectious individuals. Elife.

[25] Mohlendick B (2021). ACE2 polymorphism and susceptibility for SARS-CoV-2 infection and severity of COVID-19. Pharmacogenet. Genom..

[26] Nikiforuk AM (2021). The contrasting role of nasopharyngeal angiotensin converting enzyme 2 (ACE2) transcription in SARS-CoV-2 infection: A cross-sectional study of people tested for COVID-19 in British Columbia, Canada. EBioMedicine.

[27] Wang R (2021). Genetic screens identify host factors for SARS-CoV-2 and common cold coronaviruses. Cell.

[28] Amodio E (2020). SARS-CoV-2 viral load, IFNlambda polymorphisms and the Course of COVID-19: An observational study. J. Clin. Med..

[29] Trypsteen W, Van Cleemput J, Snippenberg WV, Gerlo S, Vandekerckhove L (2020). On the whereabouts of SARS-CoV-2 in the human body: A systematic review. PLoS Pathog..

[30] Phillips MC, Quintero D, Wald-Dickler N, Holtom P, Butler-Wu SM (2022). SARS-CoV-2 cycle threshold (Ct) values predict future COVID-19 cases. J. Clin. Virol..

[31] Aranha C, Patel V, Bhor V, Gogoi D (2021). Cycle threshold values in RT-PCR to determine dynamics of SARS-CoV-2 viral load: An approach to reduce the isolation period for COVID-19 patients. J. Med. Virol..

[32] Team, C. C.-R. SARS-CoV-2 B.1.1.529 (Omicron) Variant - United States, December 1–8, 2021. *MMWR Morb. Mortal Wkly. Rep*. **70**, 1731–1734. 10.15585/mmwr.mm7050e1 (2021). 10.15585/mmwr.mm7050e1 PMC8675659 34914670

[33] Fan G, Qin X, Streblow DN, Hoyos CM, Hansel DE (2021). Comparison of SARS-CoV-2 PCR-based detection using saliva or nasopharyngeal swab specimens in asymptomatic populations. Microbiol. Spectr..

[34] Chu DKW (2020). Molecular diagnosis of a novel coronavirus (2019-nCoV) causing an outbreak of pneumonia. Clin. Chem..

[35] Pfister R, Schwarz KA, Janczyk M, Dale R, Freeman JB (2013). Good things peak in pairs: A note on the bimodality coefficient. Front. Psychol..

[36] Meyer, D., Dimitriadou, E., Hornik, K., Weingessel, A. & Leisch, F. in *Misc Functions of the Department of Statistics, Probability Theory Group (Formerly E1071), TU Wien.*

[37] Scrucca LFM, Murphy TB, Raftery AE (2016). mclust 5: Clustering, classification and density estimation using Gaussian finite mixture models. R J..

