# Supplementary material for: Bimodal distribution pattern associated with the PCR cycle threshold (Ct) and implications in COVID-19 infections
Source: Sci Rep. 2022 Aug 25;12:14544. doi: 10.1038/s41598-022-18735-2 (PMC9406279; doi:10.1038/s41598-022-18735-2)

Bimodal Distribution Pattern Associated with the PCR Cycle Threshold (*Ct*) and Implications in COVID-19 Infections

Doris Yang[1]

Donna E. Hansel[1]

Marcel E. Curlin[2]

John M. Townes[2]

William B. Messer[2,3]

Guang Fan[1]

Xuan Qin[1]*

[1]Department of Pathology & Laboratory Medicine, [2]Department of Medicine, Division of Infectious Diseases, [3]Department Molecular Microbiology and Immunology,

Oregon Health & Science University School of Medicine, Portland, Oregon 97239

***Corresponding Author:**

Xuan Qin, PhD, D(ABMM)

Department of Pathology & Laboratory Medicine

Oregon Health & Science University

3181 SW Sam Jackson Park Road, L-113

Portland, OR 97239

Phone: 503-494-5154

Fax: 503-494-6787

Email: qinxu@ohsu.edu

Supplemental Figure 1. PCR *Ct* distribution of N and ORF1ab targets associated with samples predicated to be omicron with SGTF by Fisher PCR multiplex chemistry.

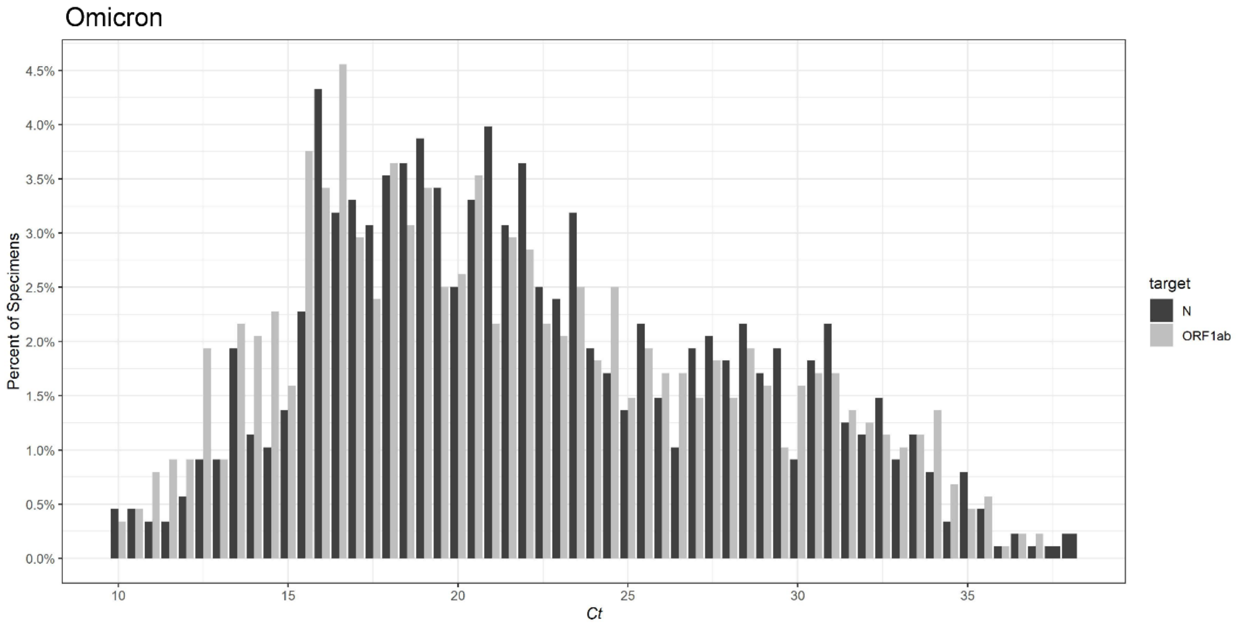

Supplemental figure 2. (a) PCR *Ct* distribution of N1 and N2 targets by CDC chemistry collected from patients of age less than 12 years (b) PCR *Ct* distribution of N1 and N2 targets by CDC chemistry collected from patients of age less than 17 years (c) PCR *Ct* distribution of N1 and N2 targets by CDC chemistry collected from patients of age less than 21 years.

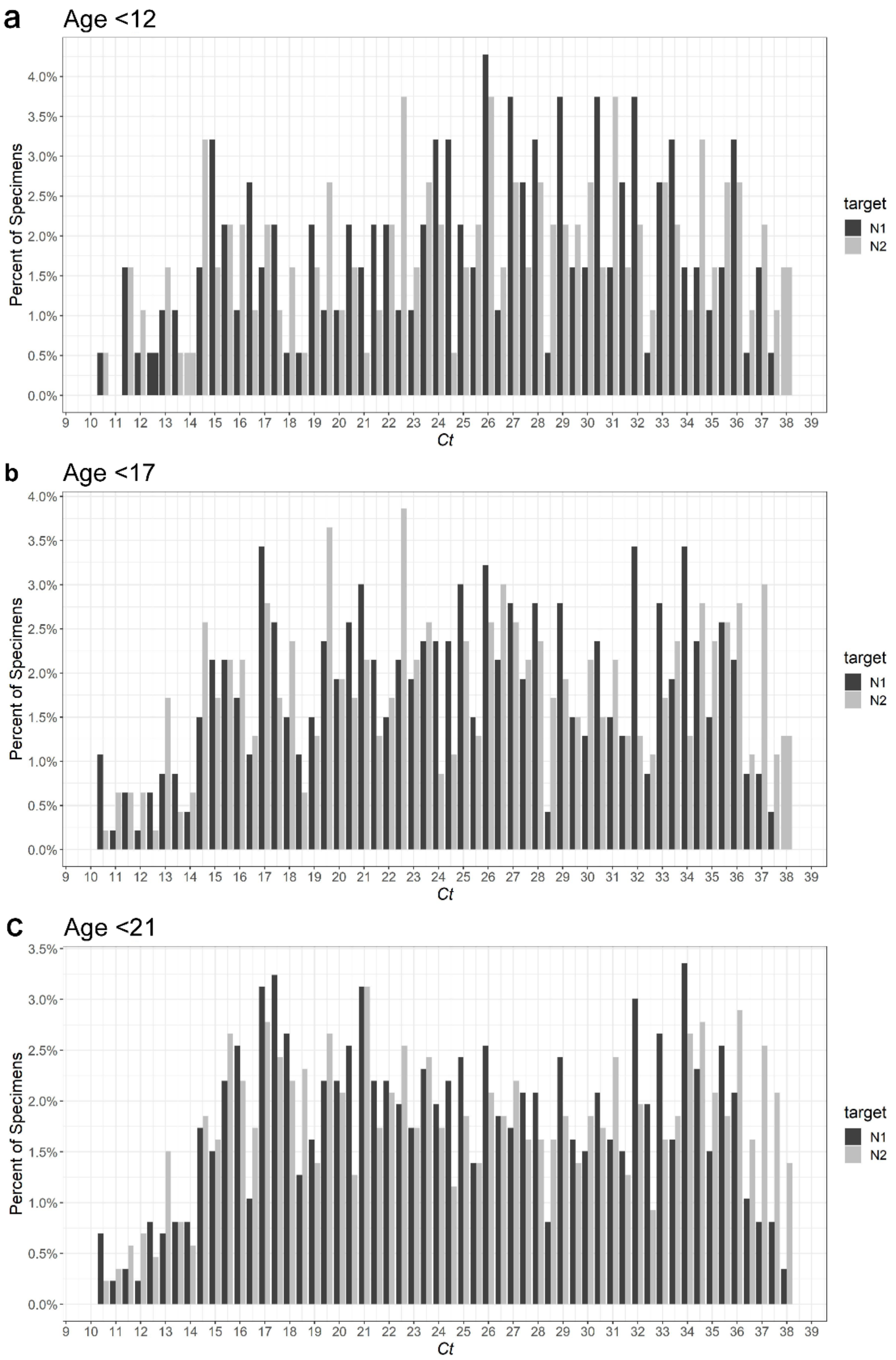

Supplement: Supplementary file 1 — Supplementary Figures. [file 41598_2022_18735_MOESM1_ESM.pdf]
